# Efficient production of secretory *Streptomyces clavuligerus* β-lactamase inhibitory protein (BLIP) in *Pichia pastoris*

**DOI:** 10.1186/s13568-018-0586-3

**Published:** 2018-04-20

**Authors:** Kin-Ho Law, Man-Wah Tsang, Yuk-Ki Wong, Ming-San Tsang, Pui-Yee Lau, Kwok-Yin Wong, Kwok-Ping Ho, Yun-Chung Leung

**Affiliations:** 1 Department of Applied Biology and Chemical Technology and the State Key Laboratory of Chirosciences, The Hong Kong Polytechnic University, Hung Hom, Kowloon, Hong Kong China; 2 Lo Ka Chung Centre for Natural Anti-Cancer Drug Development, The Hong Kong Polytechnic University, Hung Hom, Kowloon, Hong Kong China

**Keywords:** β-Lactamase inhibitor, β-Lactamase inhibitory protein, *Pichia pastoris*, Recombinant protein expression, Secretory protein expression

## Abstract

β-Lactamase inhibitory protein (BLIP), a low molecular weight protein from *Streptomyces clavuligerus*, has a wide range of potential applications in the fields of biotechnology and pharmaceutical industry because of its tight interaction with and potent inhibition on clinically important class A β-lactamases. To meet the demands for considerable amount of highly pure BLIP, this study aimed at developing an efficient expression system in eukaryotic *Pichia pastoris* (a methylotrophic yeast) for production of BLIP. With methanol induction, recombinant BLIP was overexpressed in *P. pastoris* X-33 and secreted into the culture medium. A high yield of ~ 300 mg/L culture secretory BLIP recovered from the culture supernatant without purification was found to be > 90% purity. The recombinant BLIP was fully active and showed an inhibition constant (*K*_i_) for TEM-1 β-lactamase (0.55 ± 0.07 nM) comparable to that of the native *S. clavuligerus*-expressed BLIP (0.5 nM). Yeast-produced BLIP in combination with ampicillin effectively inhibited the growth of β-lactamase-producing Gram-positive *Bacillus*. Our approach of expressing secretory BLIP in *P. pastoris* gave 71- to 1200-fold more BLIP with high purity than the other conventional methods, allowing efficient production of large amount of highly pure BLIP, which merits fundamental science studies, drug development and biotechnological applications.

**Electronic supplementary material:**

The online version of this article (10.1186/s13568-018-0586-3) contains supplementary material, which is available to authorized users.

## Introduction

β-Lactamase inhibitory protein (BLIP) is a low molecular weight protein (~ 17.5 kDa) naturally secreted by gram-positive bacterium *Streptomyces clavuligerus* (Doran et al. 1990). As its name suggests, BLIP can inhibit β-lactamases, which are bacterial enzymes that can hydrolyze β-lactam antibiotics, leading to bacterial resistance against these antibiotics. The inhibition mechanism is based upon the non-covalent competitive binding of BLIP to β-lactamases. The concave-shaped BLIP embraces β-lactamase by inserting its β hairpin loops into the active site of β-lactamase, completely masking β-lactamase’s active site from binding and hydrolyzing the β-lactam substrates (Strynadka et al. 1996). BLIP shows differential binding affinity to and inhibitory effect on different β-lactamases (Strynadka et al. 1994). It generally demonstrates specificity toward class A β-lactamases, inactivating them with inhibition constant (*K*_i_) ranged from picomolar to micromolar (Doran et al. 1990; Rudgers et al. 2001; Zhang and Palzkill 2003; Yuan et al. 2011). In particular, it shows a potent inhibition against the clinically important TEM-1 β-lactamase with a *K*_i_ of 0.1–0.6 nM (Strynadka et al. 1994; Petrosino et al. 1999; Rudgers et al. 2001). On the contrary, BLIP does not inhibit class B, C and D β-lactamases (Strynadka et al. 1994).

Because of the nanomolar-affinity interaction between BLIP and TEM-1 β-lactamase and the potent inhibitory effect of BLIP on TEM-1 enzyme, BLIP has found its implications in various aspects. First, from the point of view of biophysics, the potent interaction between BLIP and TEM-1 β-lactamase makes BLIP an appealing study model for protein–protein interaction. Determinants for the strong binding of BLIP with TEM-1 β-lactamase have been extensively characterized to elucidate the general principles of affinity and specificity in protein–protein interaction (Strynadka et al. 1996; Selzer et al. 2000; Zhang and Palzkill 2003; Kozer et al. 2007; Wang et al. 2007; Cohen-Khait and Schreiber 2016). Second, BLIP has its therapeutic value as a proteinaceous β-lactamase inhibitor. Because of the susceptibility of β-lactam antibiotics toward the degradation by β-lactamases, small-molecule β-lactamase inhibitors are often co-administrated with β-lactam antibiotics to inactivate β-lactamases, thus protecting the antibiotics from the hydrolysis by β-lactamases. Augmentin (amoxillicin and clavulanic acid) and AVYCAZ^®^ (ceftazidime and avibactam) are two examples of the formulation of β-lactam antibiotic/inhibitor used in the current clinical settings (Stein and Gurwith 1984; Zhanel et al. 2013). With the rapid emergence of β-lactamase-mediated antibiotic resistance, there is a pressing need to identify novel β-lactamase inhibitors, either natural or synthetic compounds, to restore the efficacy of the β-lactam antibiotics that are susceptible to the hydrolytic action of the newly emerged β-lactamases (Meziane-Cherif and Courvalin 2014). Regarding the inhibitory effect of BLIP on clinically prominent class A β-lactamases, BLIP has a high potentiality to become a protein drug that is co-formulated with β-lactam antibiotics in order to allow effective treatment strategy for bacterial infections. Attempts have been made to develop peptide drugs derived from the critical components of BLIP that are involved in the binding to TEM-1 β-lactamase for the inactivation of β-lactamases (Rudgers et al. 2001; Rudgers and Palzkill 2001; Sun et al. 2005; Alaybeyoglu et al. 2015, 2017). In addition, it is envisioned that by mutating BLIP, BLIP may turn into a tight binder to other classes of β-lactamases, effectively inactivating these β-lactamases (Strynadka et al. 1996; Huang et al. 1998; Rudgers and Palzkill 1999; Huang et al. 2000; Wang et al. 2007; Yuan et al. 2011; Chow et al. 2016). Third, the property of high-affinity binding of BLIP to TEM-1 β-lactamase has been recently adapted to a variety of biotechnological applications (Khait and Schreiber 2012; Banala et al. 2013; Janssen et al. 2015; Hu et al. 2016).

In order to have sufficient amount of highly purified BLIP for the above purposes, it is important to have an efficient system for the production of BLIP. So far, various approaches have been reported to obtain BLIP (Table 1). Extraction of BLIP from its native host *S. clavuligerus* and heterologous expression of BLIP using another *Streptomyces* species, *S. lividian*, gave a limited quantity of BLIP (Doran et al. 1990; Paradkar et al. 1994), suggesting that *Streptomyces* may not be optimal for over-producing BLIP. Production of BLIP as a heterologous recombinant protein in the well-established *E. coli* expression system has been reported to allow improved yield of BLIP (~ 0.25 mg to ~ 4.2 mg/L culture of BLIP) (Albeck and Schreiber 1999; Petrosino et al. 1999; Rudgers and Palzkill 1999; Reyonlds et al. 2006; Hu et al. 2016). The high expression level of recombinant BLIP in *E. coli* might be due to the high copy number of expression plasmid and the use of strong promoter for inducing protein expression. In addition, production of BLIP as a histidine-tagged protein in *E. coli* greatly simplified the subsequent purification strategy, minimizing the protein loss resulted from multiple steps of purification (Petrosino et al. 1999; Hu et al. 2016). However, BLIP formed inclusion bodies when being expressed in *E. coli* and this required the use of denaturing agents (e.g. urea) to solubilize the inclusion bodies prior to purification (Albeck and Schreiber 1999; Hu et al. 2016). To circumvent this constrain, addition of signal peptide sequence into the upstream of the *blip* gene in the expression construct was employed to direct the translated BLIP protein into the periplasmic space of *E. coli* (Petrosino et al. 1999; Reyonlds et al. 2006). Furthermore, the strategy of expressing BLIP in a secretory fashion in *B. subtilis*, which yielded ~ 3.5 mg/L culture of BLIP, was developed (Liu et al. 2004).

**Table 1:** Production of purified BLIP using various expression systems

Expression host	Mode of expression	Purification steps involved	Yield of BLIP	References
*Streptomyces clavuligerus* (Native host)	Secretory protein with BLIP’s own signal peptide	(1) Ammonium sulfate precipitation (2) Gel filtration (3) Ion exchange chromatography	BLIP represented 10% of the total exocellular proteins in *S. clavuligerus* 1.24 mg from 37.4 mg total protein of culture filtrate	Doran et al. (1990)
*Streptomyces lividans*	Secretory protein with native BLIP signal peptide	Not purified	Amount in culture filtrate was 12-fold lower than that in *Streptomyces clavuligerus*	Paradkar et al. (1994)
*Escherichia coli*	Intracellular fusion protein with maltose-binding protein	Not reported	Not reported	Rudgers and Palzkill (1999)
	Intracellular protein that formed inclusion bodies	(1) Ion exchange chromatography (2) Gel filtration	~ 1.6–4.2 mg/L culture	Albeck and Schreiber (1999)
	Intracellular 6×His-tag protein that formed inclusion bodies	Metal affinity chromatography	2.2 mg/L culture	Hu et al. (2016)
	Intracellular 6×His-tag protein with β-lactamase signal peptide that was transported to the periplasmic space	Metal affinity chromatography	~ 0. 25 mg/L culture	Petrosino et al. (1999)
	Intracellular protein with native *S. clavuligeris* signal peptide that was transported to the periplasmic space	(1) Ion exchange chromatography (2) Gel filtration	0.5 mg/L culture	Reyonlds et al. (2006)
*Bacillus subtilis*	Secretory protein with native BLIP signal peptide	(1) Ammonium sulfate precipitation (2) Ion exchange chromatography	~ 3.5 mg/L culture	Liu et al. (2004)
*Pichia pastoris*	Secretory protein with α-factor mating signal peptide	Not purified	~ 300 mg/L culture	This study

In this study, we devised a secretory expression system in *Pichia pastoris* for high-level production of BLIP. *P. pastoris* is a methylotrophic yeast which utilizes methanol (MeOH) as a sole carbon source. It has been an expression host optimized for high-level expression for foreign proteins in either the intracellular or secretory modes (Cereghino and Cregg 2000; Ahmad et al. 2014; Krainer et al. 2016). Here, we subcloned the *blip* gene from *S. clavuligerus* to a commercially available plasmid pPICZαA for secretory protein expression in *P. pastoris*. Our results demonstrate that under the control of methanol-inducible promoter of the alcohol oxidase 1 (AOX1) gene and signal peptide processing of the translated protein, BLIP can be successfully expressed in *P. pastoris* in a secretory manner. Unprecedentedly large amount of ~ 300 mg BLIP with high purity was obtained directly from the supernatant of 1 L culture (Table 1). The recombinant BLIP was functionally active and showed same *K*_*i*_ as the native BLIP isolated from *S. clavuligerus* did. Furthermore, the *Pichia*-produced BLIP enhanced the bacteria killing efficiency of ampicillin (a penicillin-type of β-lactam antibiotic) in β-lactamase-producing Gram-positive *B. subtilis*.

## Materials and methods

### Bacterial strains, plasmids and chemicals

*Streptomyces clavuligerus* (ATCC 27064) was purchased from ATCC (Manassas, VA, USA). *E. coli* XL1-Blue for transformation was obtained from lab stock. *P. pastoris* X-33 and plasmid pPICZαA were obtained from Invitrogen (Carlsbad, CA, USA). *B. subtilis* 168 haboring plasmid pYCL18 and *E. coli* haboring pRSET-K/TEM-1 β-lactamase were from lab stock. Ampicillin and chloramphenicol were purchased from Sigma (St. Louis, MO, USA) whereas Zeocin was from Invitrogen (Carlsbad, CA, USA).

### Preparation of chromosomal DNA from *S. clavuligerus*

*Streptomyces clavuligerus* was streaked on a nutrient agar plate. A single colony of *S. clavuligerus* was inoculated to a 5 mL of LB medium and cultivated at 30 °C with shaking for 2–3 days. Chromosomal DNA from *S. clavuligerus* was extracted from 1 mL of the inoculum by Wizard Genomic DNA purification kit (Promega, Madison, WI, USA) according to manufacturer’s manual (Technical Manual No. 050, Promega).

### Subcloning of *blip* gene into expression plasmid pPICZαA

Amplification of *blip* gene (Accession Number: AAA16182) by polymerase chain reaction was performed by iProof DNA polymerase (Bio-Rad, Hercules, CA, USA) using the chromosomal DNA from *S. clavuligerus* as a template. A forward primer (5′-gat ata **GAA TTC** gcg ggg gtg atg acc ggg gcg-3′) and a reverse primer (5′ gat ata **TCT AGA** ggt cga ctc ctt cgg cga cg-3′), containing *Eco*RI and *Xba*I sites (bolded) respectively, were employed to amplify the sequence of the *blip* gene encoding the mature protein with its transcription terminator. The amplified PCR product was digested by *Eco*RI and *Xba*I and then ligated with an *Eco*RI-*Xba*I double digested pPICZαA. The ligation mixture was transformed into *E. coli* XL1-Blue and the transformants were selected on low salt LB agar plates containing 25 μg/mL Zeocin. The resultant plasmid was designated as pPICZαA/BLIP.

### Transformation of expression plasmid into *P. pastoris*

Plasmid pPICZαA/BLIP was transformed into *P. pastoris* by the method of electroporation. Briefly, plasmid pPICZαA/BLIP was first digested by *Sac*I for linearization to promote the integration of the *blip* gene into the chromosome of *P. pastoris* via homologous recombination. Then 5–10 μg of linearized plasmid was mixed with the competent cells of *P. pastoris* X-33 (prepared as described in the manufacturer’s manual) in an electroporation cuvette and incubated on ice for 5 min. Electroporation was performed by using Gene Pulser II (Bio-Rad, Hercules, CA, USA), with a setting of 1.5 kV with 200 Ω, 25 μF capacitance, and a pulse time of 5–7 ms. Afterward, 1 mL of ice-cold 1 M sorbitol was added to the cuvette immediately. The mixture was then transferred to several sterile eppendorf tubes, which were incubated at 30 °C for 1.5 h. After that, it was centrifuged at 3000*g* for 1 min. Supernatant was removed and the cells were resuspended in a 200 µL of 1 M sorbitol solution. The cells were then plated on YPD agar plates containing 100 µg/mL Zeocin and incubated at 30 °C for 3 days. Several single colonies were picked from the plate and then streaked on a fresh YPD agar plate containing 800 µg/mL Zeocin for further selection for the multi-copy recombinants.

### Over-expression of recombinant BLIP in *P. pastoris*

A single colony of recombinant *P. pastoris* X-33 integrated with *blip* gene was inoculated in 5 mL of YPD medium with 100 μg/mL Zeocin and incubated at 30 °C with 250 rpm agitation for 20 h. A 0.5 mL of the overnight culture was then used to inoculate a 100 mL of BMGY (100 mM potassium phosphate, pH 6.0; 1.34% YNB; 4 × 10^−5^% biotin; 1% glycerol; 1% yeast extract; 2% peptone) in a 1 L-flask. The cells were incubated at 30 °C with a 250 rpm agitation and grown for 20 h. When OD_600_ reached more than 5.0, the cells were pelleted by centrifugation for 10 min at 3000*g*, 4 °C. The cells were then resuspended with a 20 mL of BMMY (100 mM potassium phosphate, pH 6.0; 1.34% YNB; 4 × 10^−5^% biotin; 1% yeast extract; 2% peptone) supplemented with 2% MeOH to allow methanol-induced protein expression. The culture was incubated at 30 °C with a 250 rpm agitation for 72 h. A 2% MeOH was added to the culture every 24 h so as to provide the carbon source and maintain the induction. The expression and purity of BLIP were assessed by SDS-PAGE analysis whereas total protein concentration was determined by Bradford assay.

### ESI–MS analysis

Prior to ESI–MS, the BLIP sample was desalted with Milli-Q water by an Amicon Ultra-4 centrifugal filter device (cut-off = 10,000 Da; Millipore, Bedford, MA, USA). A 10 ρmol/μL of BLIP solution in 1:1 (v/v) water-acetonitrile with 15% ammonium hydroxide was then prepared for ESI–MS. The ESI mass spectrum was obtained in the negative ion mode with a quadrupole time of flight (Q-Tof 2, Micromass, Altrincham, UK) mass spectrometer equipped with a Z-spray electrospray ionization source. Masslynx software version 4.1 was used as an operating interface for the instrument. The ESI-Q-TOF MS operating parameters were optimized and set as follows: ESI capillary voltage, 2000–3000 V; sample cone voltage, 30–50 V; source temperature, 80 °C; desolvation temperature, 150 °C; flow-rate of desolvation gas (N_2_), 350 L/h; flow-rate of cone gas (N_2_), 50 L/h. The *m/z* range of 500–3000 was monitored. The instrument was calibrated with a 10 ρmol/μL of horse heart myoglobin [in a 1:1 water-acetonitrile mixture (v/v)]. BLIP was assumed to be represented by a series of peaks corresponding to multiply protonated ions in the mass spectrum. This multiply charged mass spectrum was processed by a transform program to obtain the molecular mass of BLIP.

### *K*_i_ determination

The *K*_i_ value for the BLIP against TEM-1 β-lactamase was determined using the method described by Petrosino et al. (1999). Procedure for the production of TEM-1 β-lactamase was mentioned in Additional file 1: Methods. 1.5 nM TEM-1 β-lactamase was pre-incubated with varying concentrations of BLIP (0–15 nM) in 50 mM sodium phosphate buffer containing 1 mg/mL bovine serum albumin for 2 h at 25 °C. Nitrocefin was then added to the mixture of BLIP-TEM1 β-lactamase at a final concentration of 21 μM. Hydrolysis of nitrocefin was monitored by the increase in absorbance at the wavelength at 500 nm. The equilibrium dissociation constant (*K*_i_*) was calculated by fitting the plot of the concentrations of free β-lactamase versus concentrations of inhibitor (BLIP) with a nonlinear regression equation (Eq. 1) using the program OriginPro 6.0 (OriginLab Corporation).

1 $$ \left[ {E_{free} } \right] = \left[ {E_{0} } \right] - \frac{{\left[ {E_{0} } \right] + \left[ {I_{0} } \right] + K_{i} * - \sqrt {\left( {\left[ {E_{0} } \right] + \left[ {I_{0} } \right] + K_{i} *} \right)^{2} - \left( {4\left[ {E_{0} } \right]\left[ {I_{0} } \right]} \right)} }}{2} $$

where [*E*_*free*_] is the concentration of free β-lactamase, [*E*_0_] is the initial β-lactamase concentration, and [*I*_0_] is the initial inhibitor (BLIP) concentration. *K*_i_* is equivalent to *K*_i_, which is the inhibition constant (Petrosino et al. 1999).

### In vitro β-lactamase inhibitory assay

*Bacillus subtilis* haboring pYCL18 that constitutively co-expresses PenP and PenPC β-lactamases was cultivated in 5 mL of LB broth at 37 °C with agitation at 280 rpm. When OD_600_ reached 3.0, a 5 μL of the culture was transferred to a fresh 5 mL of LB broth with different composition of BLIP and ampicillin. The inoculums were allowed to grow at 37 °C with agitation at 280 rpm. Cell growth was monitored by measuring OD_600_ of the cell culture at different time intervals.

## Results

### Recombinant BLIP was over-expressed as a secretory protein in *P. pastoris*

**Fig. 1:**
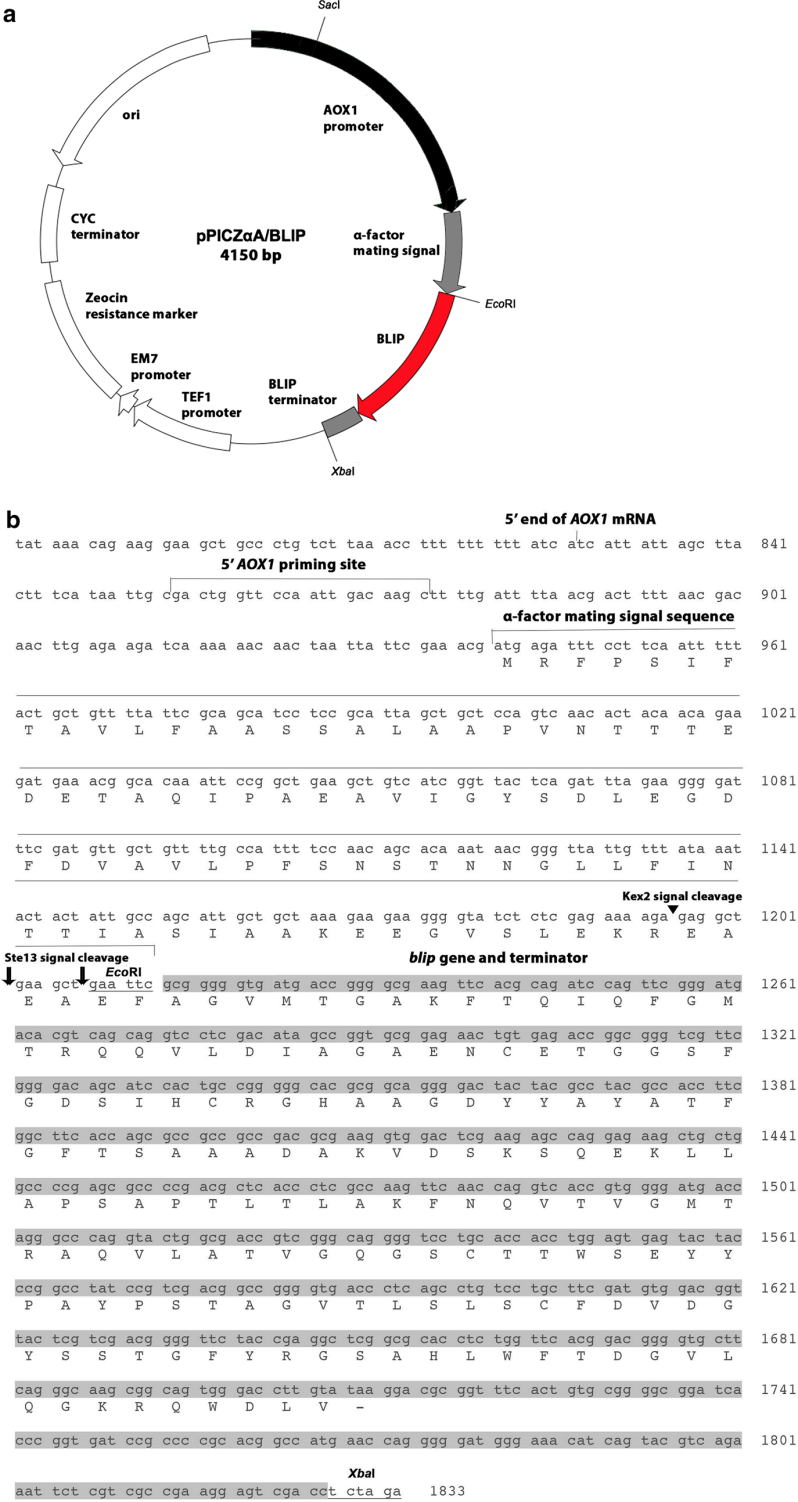
Expression construct pPICZαA/BLIP for production of secretory BLIP in *P. pastoris*. **a** Plasmid map of pPICZαA/BLIP (*AOX1 promoter* alcohol oxidase 1 promoter that permits the methanol-inducible expression of BLIP in *Pichia*, *BLIP terminator blip* transcription terminator that allows 3′mRNA processing of *blip* gene, *TEF1 promoter and EM7 promoter* transcription elongation factor 1 gene from *Saccharomyces cerevisiae* and a synthetic prokaryotic promoter that drive the expression of the Zeocin resistance gene, *Zeocin resistance marker* Sh ble gene1 whose product confers resistance to Zeocin in *Pichia* cells for selection, *CYC terminator* 3′ end of the *Saccharomyces cerevisiae* cytochrome c1 gene that allows efficient 3′ mRNA processing of the Zeocin resistance gene, *ori* origin of replication); **b** Sequence that encodes the mature protein of BLIP was placed downstream the AOX1 promoter and α-factor mating signal sequence (sequence encoding mature BLIP and the *blip* transcription terminator were shaded; *Eco*RI and *Xba*I restriction sites were underlined; Kex2 and Ste13 signal cleavage sites were indicated by arrow head and arrow respectively)

**Fig. 2:**
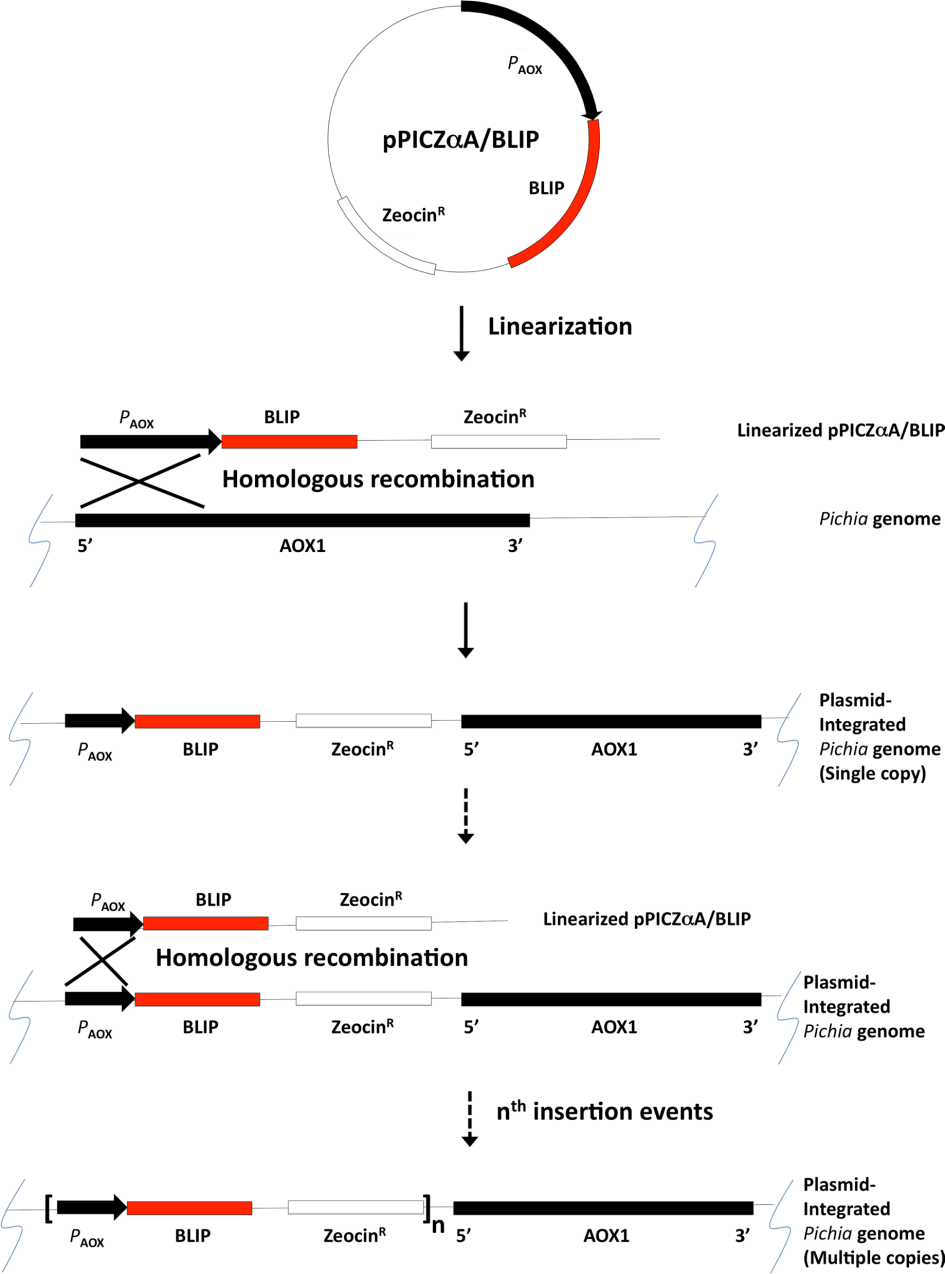
Integration of pPICZαA/BLIP into genome of *P. pastoris* X-33 via homologous recombination. (*P*_*AOX*_ AOX1 promoter, *BLIP* expression cassette of BLIP including the α-factor signal sequence, *blip* gene and *blip* transcription terminator, *Zeocin*^*R*^ Zeocin resistance gene, *AOX* AOX1 gene)

To construct the recombinant yeast strain for expression of BLIP, an expression vector was constructed by inserting the *blip* gene and its terminator sequence from *S. clavuligerus* into the pPICZαA at the region downstream the AOX1 promoter and α-factor mating signal sequence (Fig. 1a, b). The resultant plasmid pPICZαA/BLIP was then *Sac*I-linearized and transformed into *P. pastoris* X-33. During transformation, the *blip* gene was integrated into the *Pichia* genome via homologous recombinant at AOX1 locus either as a single copy or multiple copies (Fig. 2). Integrants were then selected with Zeocin. The selected recombinant *Pichia* integrant was ready for heterologous expression for BLIP.

**Fig. 3:**
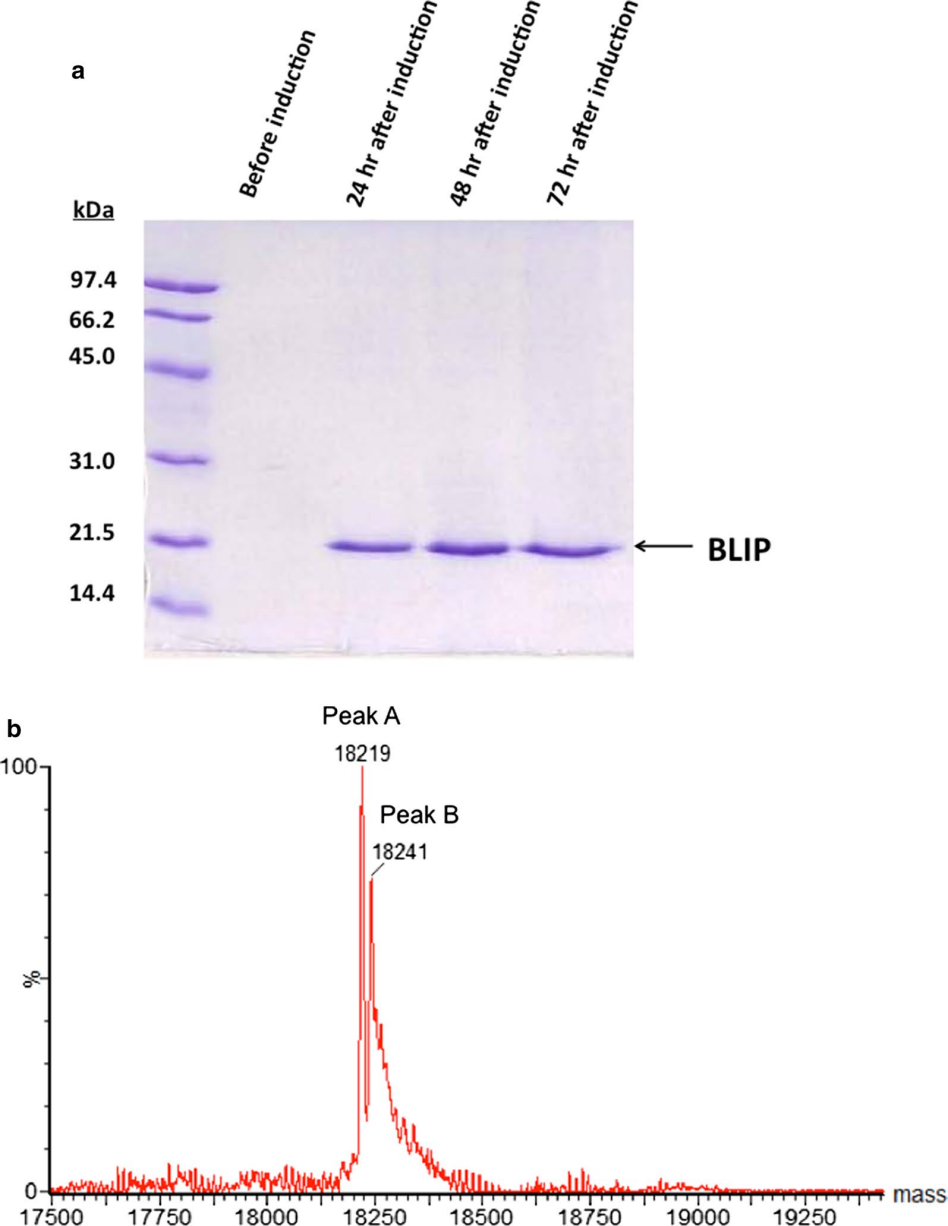
Expression of secretory BLIP in *P. pastoris*. **a** As revealed by a coomassie-blue stained SDS-PAGE gel, highly pure BLIP was found to be present in the culture media collected 24, 48 and 72 h after induction with 2% MeOH. **b** As analyzed by ESI–MS, the measured mass of the secretory BLIP (peak A) was 18,219 which matched the calculated mass of EAEAEF-mature BLIP (18,219.25). Peak B with molecular mass of 18,241 corresponded to the sodium adduct of the secretory BLIP

Recombinant *P. pastoris* cells with *blip* gene were first cultivated in BMGY medium for 20 h. To induce the expression of the target protein (BLIP), the cells were collected and transferred to BMMY containing 2% MeOH and then allowed to grow for further 72 h. According to our data, there was no BLIP detected in the culture medium before the methanol-induction, indicating that the expression of BLIP was tightly controlled by the AOX1 promoter in *P. pastoris* (Fig. 3a). Secretory BLIP was found in the culture supernatants collected at 24, 48 and 72 h after methanol-induction. Approximately 300 mg of > 90% pure BLIP/L culture was, in total, recovered from the culture supernatant.

The secretory BLIP obtained from the culture of *P. pastoris* was analyzed by ESI–MS (Fig. 3b). The measured mass of BLIP was 18219, which corresponds to the calculated mass of mature BLIP with a peptide of EAEAEF at its N-terminus (18,219.25). The results suggest that the part of the signal peptide of the pro-protein of *P. pastoris*-expressed BLIP was cleaved at the site between Arg and Glu by the aminopeptidase Kex2 protease to release the EAEAEF-mature BLIP and further trimming of the amino-terminal Glu-Ala residue repeats by the *STE*13 gene product did not occur (Fig. 1b).

### *P. pastoris*-expressed BLIP showed tight binding with TEM-1 β-lactamases

**Fig. 4:**
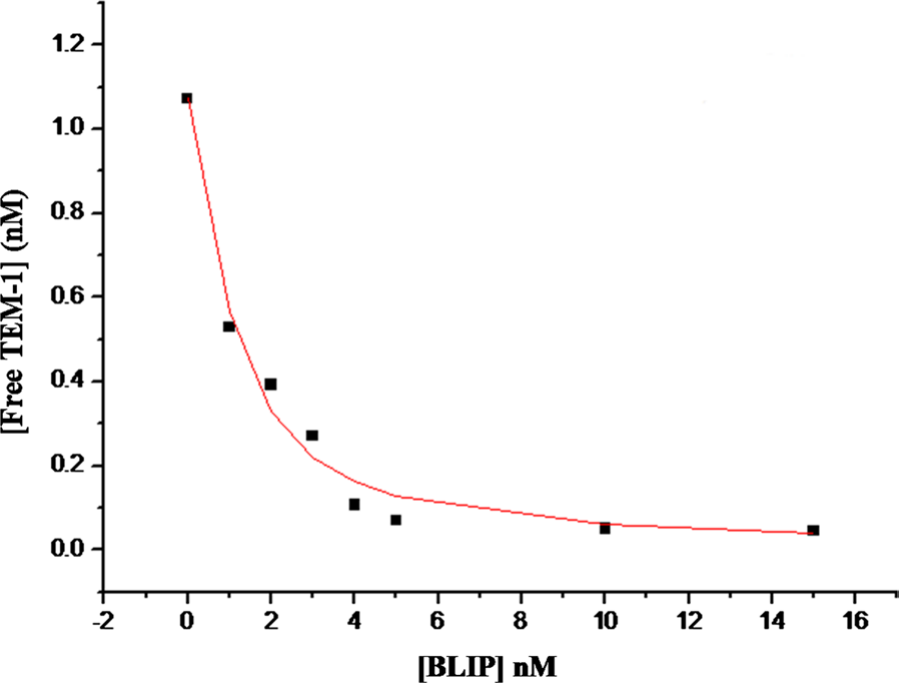
Determination of the *K*_i_ value of the *P. pastoris*-expressed BLIP against the TEM-1 β-lactamase. 1.5 nM TEM1 β-lactamase was pre-incubated with varying concentrations of BLIP (0–15 nM) in 50 mM sodium phosphate buffer containing 1 mg/mL bovine serum albumin for 2 h at 25 °C. Remaining concentration of free β-lactamase at varying concentrations of BLIP was then estimated by the spectrometric β-lactamase assay using nitrocefin as a substrate. The plot of concentrations of free β-lactamase versus varying amount of BLIP represents the nonlinear regression fit of the data to Eq. (1) for the *K*_i_ calculation using the program OriginPro 6.0. Each point represents a single measurement. The experiment was repeated in duplicate. The determined *K*_i_ was 0.55 ± 0.07 nM

To assess the binding ability of the *P. pastoris*-expressed BLIP to TEM-1 β-lactamase, the inhibition constant (*K*_i_) value of BLIP on TEM-1 β-lactamase was evaluated. Recombinant BLIP expressed in *P. pastoris* exhibited a *K*_i_ of 0.55 nM (Fig. 4). This *K*_i_ value was comparable to the reported *K*_i_ value (0.5 nM) of the native BLIP from *S. clavuligerus*. This indicated that the secreted BLIP from *P. pastoris* was correctly folded and showed similar performance as the *S. clavuligerus*-expressed BLIP in term of the association with TEM-1 β-lactamase.

### Co-administration of ampicillin with BLIP inhibited growth of β-lactamases-producing *B. subtilis*

**Fig. 5:**
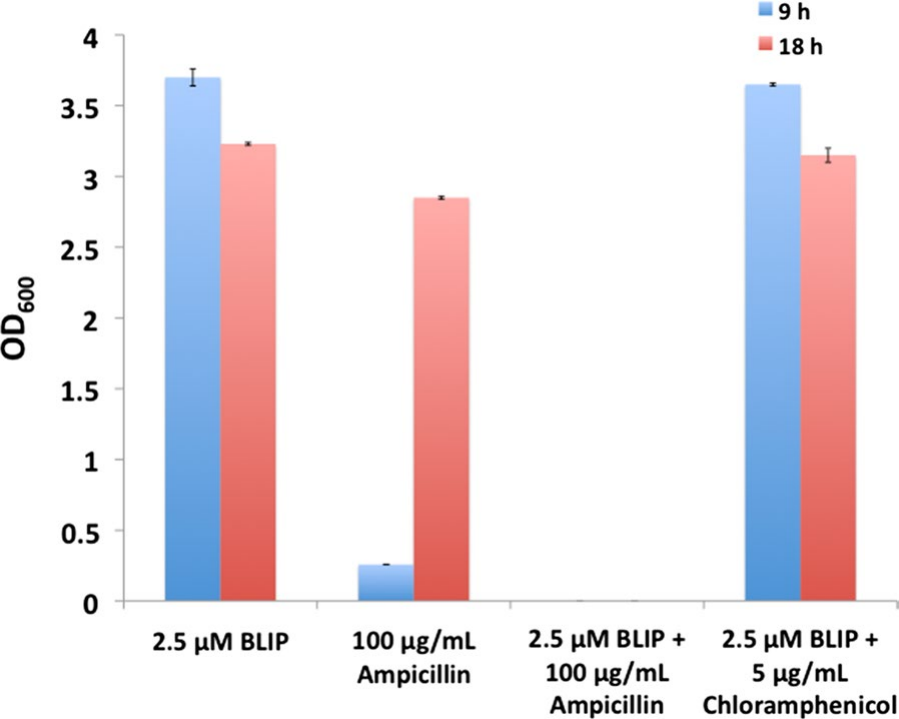
Antimicrobial effect of BLIP with ampicillin on β-lactamase-producing *B. subtilis*. A strain of *B. subtilis* haboring pYCL18 was cultivated in LB broth at 37 °C with shaking at 280 rpm in the presence of: (1) 2.5 μM BLIP; (2) 100 μg/mL ampicillin; (3) 2.5 μM BLIP and 100 μg/mL ampicillin; and (4) 2.5 μM BLIP and 5 μg/mL chloramphenicol. The bacterial growth of the cultures was monitored by OD_600_ at time intervals of 9 and 18 h. The experiment was repeated in duplicate

To test the β-lactamase inhibitory effect of BLIP on bacterial growth, BLIP was added to the culture of a genetically modified Gram-positive *B. subtilis* strain (*B. subtilis*/pYCL18) that constitutively secretes PenP and PenPC β-lactamases (Gray and Chang 1981; Madgwick and Waley 1987). *B. subtilis*/pYCL18 can grow in the LB broth supplemented with 100 μg/mL ampicillin owing to the resistance conferred by β-lactamases (Fig. 5). Apart from this, the strain of *B. subtilis*/pYCL18 is also resistant to chloramphenicol due to the presence of a chloramphenicol acetyltransferase (*cat*) gene in pYCL18 (Additional file 1: Figure S1). BLIP itself has no bacterial killing effect and showed no inhibitory effect on bacterial growth in our preliminary study (data not shown). Addition of 2.5 μM BLIP with 100 μg/mL ampicillin exerted an antimicrobial effect in which cannot be observed from the cultures that were added with either 2.5 μM BLIP only or 2.5 μM BLIP with 5 μg/mL chloramphenicol (Fig. 5).

## Discussion

Considering the tight interaction between BLIP and various class A β-lactamases, BLIP is an intriguing protein not only having its importance as a study model for protein–protein interaction but also showing its potential applications in biopharmaceutical industry and biotechnology. To fulfill the needs for sufficient supply of BLIP for various purposes, it is necessary to develop highly productive system to obtain BLIP.

Our study illustrated the first time to make use of *P. pastoris* as an expression platform for producing secretory BLIP. We attempted to develop the *P. pastoris*-based production system for BLIP based on several reasons. First, *P. pastoris* has been well characterized and developed for heterologous protein expression (Cereghino and Cregg 2000; Ahmad et al. 2014). Easy genetic manipulation in *P. pastoris* favors the genetic modification of BLIP for research and biotechnological purposes. Second, *P. pastoris* has demonstrated its powerful capability to produce high level of correctly folded foreign proteins extracellularly (Cereghino and Cregg 2000; Ahmad et al. 2014). In addition, *blip* gene has a high GC-content (66%) (Doran et al. 1990). The high GC content of *blip* gene may contribute to the formation of secondary structure in the mRNA during transcription, which subsequently interrupts the translation process, leading to a low expression level of BLIP. Taken the factor of GC content into consideration, *P. pastoris* may be a favorable expression host for proteins encoded by GC rich gene (Daly and Hearn 2005) as suggested by several cases of high level expression of foreign genes with enriched GC content in *P. pastoris* (Clare et al. 1991; Olsen et al. 1996; Tull et al. 2001). Taken together, it was speculated that a high level of secretory BLIP expression might be achieved in the *Pichia* expression system. Third, regarding the potentiality of BLIP to be a biopharmaceutical, *P. pastoris* is well suited for producing BLIP for pharmaceutical use because *P. pastoris* is generally recognized as safe and various *P. pastoris*-expressed biopharmaceutical proteins have gained FDA approval (Çelik and Çalık 2012; Berlec and Strukelj 2013; Gonçalves et al. 2013; Meehl and Stadheim 2014).

From our results, a high titer of ~ 300 mg/L culture of secreted BLIP was achieved in *P. pastoris*. The recombinant BLIP was found to be highly pure (> 90%) in the culture medium and could be easily recovered by clarifying the culture medium by centrifugation. Compared with other approaches that utilize *E. coli* and *B. subtilis* expression system, giving several milligrams per L culture of BLIP (Albeck and Schreiber 1999; Petrosino et al. 1999; Reyonlds et al. 2006; Liu et al. 2004; Hu et al. 2016), our approach using *Pichia* for producing secretory BLIP showed a remarkable enhancement in the production yield of pure BLIP. In addition, secretory BLIP can be recovered directly from the culture supernatant, facilitating the downstream process for obtaining BLIP. Furthermore, since *P. pastoris* is favorable for fermentative growth due to capability to grow at high cell density (Olsen et al. 1996), the current system can be scale-up by fermentation to meet greater demands. The efficient production system of secretory BLIP using *P. pastoris* will be able to provide a promising supply of pure BLIP in large quantity, undoubtedly facilitating the study of BLIP and also the application of BLIP in pharmaceutical industry and biotechnology.

## Additional file

**Additional file 1.**

## Abbreviations

BLIP: β-lactamase inhibitory protein
*K*_i_: inhibition constant
h: hour
min: minute
s: second
rpm: revolution per minute
