# Supplementary material for: Efficient production of secretory Streptomyces clavuligerus β-lactamase inhibitory protein (BLIP) in Pichia pastoris
Source: AMB Express. 2018 Apr 20;8:64. doi: 10.1186/s13568-018-0586-3 (PMC5910447; doi:10.1186/s13568-018-0586-3)
Supplement: Supplementary file 1 — Additional file 1. [file 13568_2018_586_MOESM1_ESM.pdf]

AMB Express

Efficient production of secretory *Streptomyces clavuligerus* β-lactamase inhibitory protein (BLIP) in *Pichia pastoris* for restoring resistant Gram-positive bacterial susceptibility to β-lactam antibiotics

Kin-Ho Law[1,†], Man-Wah Tsang[1,†], Yuk-Ki Wong[1], Ming-San Tsang[1], Pui-Yee Lau[1], Kwok-Yin Wong[1], Kwok-Ping Ho[1,*], Yun-Chung Leung[1,2,*]

[1]Department of Applied Biology and Chemical Technology and the State Key Laboratory of Chirosciences, The Hong Kong Polytechnic University, Hung Hom, Kowloon, Hong Kong, China
[2]Lo Ka Chung Centre for Natural Anti-Cancer Drug Development, The Hong Kong Polytechnic University, Hung Hom, Kowloon, Hong Kong, China

[†]These authors contributed equally to this work.

***Correspondence:** Prof. Yun-Chung Leung, Department of Applied Biology and Chemical Technology, The Hong Kong Polytechnic University, Hung Hom, Kowloon, Hong Kong, China
E-mail: thomas.yun-chung.leung@polyu.edu.hk

***Additional correspondence:** Dr. Kwok-Ping Ho, Department of Applied Biology and Chemical Technology, The Hong Kong Polytechnic University, Hung Hom, Kowloon, Hong Kong, China
E-mail: kwok-ping.ho@connect.polyu.edu.hk

Methods. Production of TEM-1 β-lactamase

A single colony of *E. coli* BL21(DE3) harboring pRSET-K/TEM-1 β-lactamase was inoculated into 5 mL of 2×TY medium with 50 μg/mL kanamycin and cultivated at 37°C for 14 – 16 h with shaking at 250 rpm. After that, 1 mL of the pre-culture was transferred into 100 mL of 2×TY medium and incubated at 37°C with shaking at 250 rpm. When OD_{600} of the culture was 0.8, 0.3 mM IPTG was added to the culture to induce the expression of TEM-1 β-lactamase. The culture was then incubated at 37°C with shaking at 250 rpm for an additional of 4 h. Afterward, the cells were collected by centrifugation at 10,000*g* at 4°C for 30 min. The cells were resuspended in 10 mL of solublization buffer (20 mM Tris-HCl, 0.2 M sodium chloride, pH 7.4) with 75 μg/mL lysozyme and incubated at 30°C for 45 min. Then the cells were lysed by sonication. Bacterial cell lysate was then subjected to centrifugation at 18,000*g* for 30 min at 4°C. The cell lysate was loaded onto a 5 mL of HiTrap Chelating column charged with nickel for purification. Histidine-tagged TEM-1 β-lactamase was eluted with a linear gradient elution from 0 – 0.5 M imidazole in 20 mM sodium phosphate buffer (pH 7.4). Fractions containing TEM-1 β-lactamase were pooled, dialyzed against 10 mM ammonium bicarbonate and lyophilized.

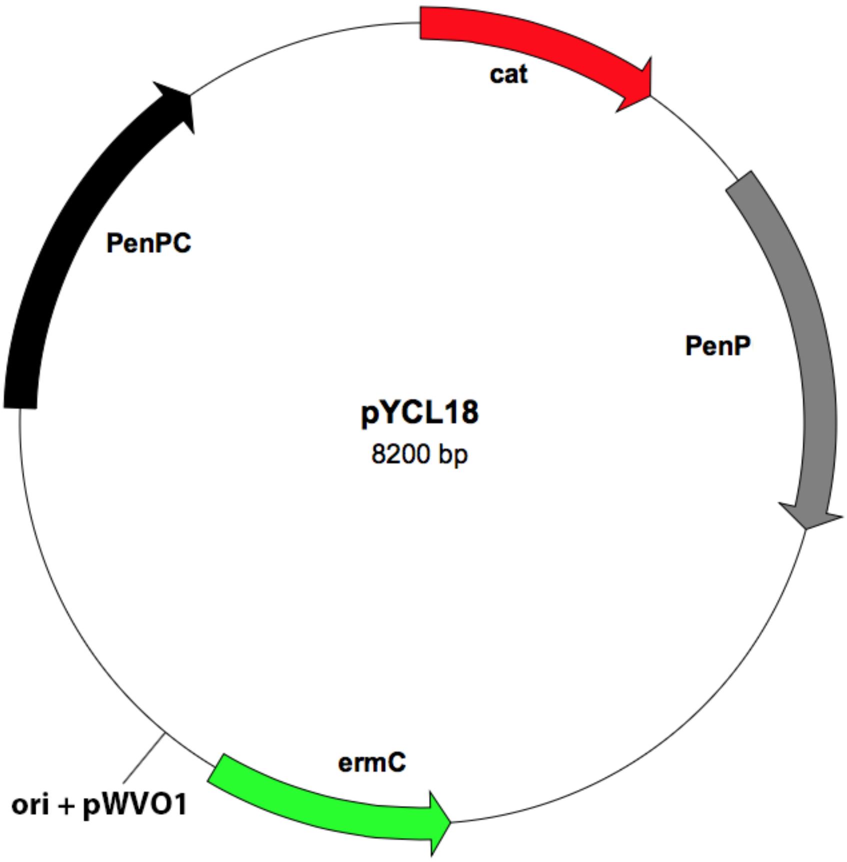

Figure. S1. Plasmid map of the *E. coli/B. subtilis* shuttle plasmid pYCL18. *cat* chloramphenicol acetyltransferase gene, *ermC* Erythromycin resistant marker, *PenP* β-lactamase gene from *Bacillus licheniformis*, *PenPC* β-lactamase gene from *Bacillus cereus*, and *ori + pWVO1* The plus origin of replication of pWWO1, a plasmid that replicates via a rolling-circle mode of replication.
